# Isolation, purification, and structural elucidation of *Stropharia rugosoannulata* polysaccharides with hypolipidemic effect

**DOI:** 10.3389/fnut.2022.1092582

**Published:** 2022-12-16

**Authors:** Yinlu Gao, Gulijiannaiti Abuduaini, Chenhe Yang, Shanshan Zhang, Yanrong Zhang, Hongxiu Fan, Xu Teng, Chenligen Bao, Hongcheng Liu, Dawei Wang, Tingting Liu

**Affiliations:** ^1^School of Food Science and Engineering, Jilin Agricultural University, Changchun, China; ^2^Scientific Research Base of Edible Mushroom Processing Technology Integration, Ministry of Agriculture and Rural Affairs, Changchun, China; ^3^Engineering Research Center of Grain Deep-Processing and High-Efficiency Utilization of Jilin, Changchun, China; ^4^Key Laboratory of Technological Innovations for Grain Deep-Processing and High-Efficiency Utilization of By-Products of Jilin, Changchun, China

**Keywords:** *Stropharia rugosoannulata*, polysaccharides, hypolipidemic, antioxidation, structure

## Abstract

*Stropharia rugosoannulata* is a widely grown edible mushroom with a high nutritional value. *S. rugosoannulata* polysaccharides is one of the most important bioactive components of *S. rugosoannulata* and has a wide range of activities. A *S. rugosoannulata* polysaccharides, named SRF-3, was derived from the *S. rugosoannulata* extraction by freeze-thaw combine with hot water extraction method, then prepareed with DEAE-cellulose column and Sephacryl S-200 HR gel column, and its hypolipidemic activity was determined. The structural characteristics of SRF-3 were analyzed by infrared spectral scanning (FT-IR), ultra-high performance liquid chromatography (UHPLC), acid hydrolysis, methylation analysis, nuclear magnetic resonance (NMR), and Gas Chromatography-Mass Spectrometer (GC-MS). SRF-3 is composed of mannose, galactose, methyl galactose and fructose with ratios of 16, 12, 58 and 12, respectively. In addition, the average relative molecular mass of SRF-3 is approximately 24 kDa. The main chain of SRF-3 is mainly composed of repeating α-D-1,6-Gal*p* and α-D-1,6-Me-Gal*p* units, with branches in the O-2 position of Gal. The structure is presumed to be a mannogalactan, with a small amount of t-β-D-Man*p* present as a side chain. Hypolipidemic activity assay showed that SRF-3 had good antioxidant and hypolipidemic effects *in vitro*, suggesting that SRF-3 have potential application in reducing liver fat accumulation.

## 1 Introduction

Edible fungi is a general term for large fungi that people can eat. More than 120,000 species of fungi have been described in the world, and more than 6,000 species can form large seed entities or mycorrhizal tissue, and more than 2,000 species are available for consumption (1, 2).

Mushrooms have a long history of consumption and are nutritious, tasty and high in protein, low in fat, essential amino acids, minerals, vitamins, and polysaccharides, making them a “healthy food” (3–6). In addition, mushrooms are unique in their nutritional value as they ensure the body’s need for non-saturated fatty acids and prevent the harmful effects of too much saturated fatty acids (7–9). They are also known for their ability to lower blood cholesterol and treat high blood pressure (10–13), while *Lentinula edodes*, *Flammulina velutipes*, and *Hericium erinaceus* contain substances that enhance the body’s ability to fight cancer (14–17).

Polysaccharides are water-soluble natural polymers composed of more than 10 monosaccharide units, and are one of the basic substances to maintain the normal life activities of the body. Polysaccharides are also commonly found in edible mushrooms, which are gradually being recognized as healthy food and medicine (18). Many studies have confirmed that edible mushroom polysaccharides are not only anti-aging and antioxidant (19, 20), but also have immunomodulatory and anti-tumor effects, in addition to regulating blood lipids, lowering cholesterol, protecting the liver and detoxifying the body, and preventing obesity and diabetes (19–28).

*Stropharia rugosoannulata* is native to Europe and the United States, but is now widely grown around the world and is one of the top 10 most traded species on the international edible mushroom market. It is rich in nutrients, with over 65% high quality carbohydrates, and studies in recent years have shown that in addition to its nutritional value, mushrooms are also effective in preventing coronary heart disease, aiding digestion, and relieving mental fatigue (29, 30).

However, few studies have been reported on the isolation and purification, structure and lipid-lowering efficacy of the polysaccharides from *S. rugosoannulata*. Therefore, the aim of this study was to extract new polysaccharides from *S. rugosoannulata* and to investigate their physicochemical properties and structure. Its potential as a lipid-lowering agent was investigated by *in vitro* antioxidant assays and *in vitro* lipid-lowering assays.

## 2 Materials and methods

### 2.1 Materials

*Stropharia rugosoannulata* was obtained from the Jilin Institute of Agricultural Science (Changchun, China) and harvested in September 2021 in Gongzhuling (Jilin Province, China). HepG2 cells were purchased and characterized at Meixuan Biological Science Co., Ltd. (Shanghai, China). Diethylaminoethyl-Sepharose Fast Flow was purchased from Shanghai Hengxin Chemical Reagent Co., Ltd. (Shanghai, China) and Sephacryl S-200 High Resolution gel (Sephacryl S-200) was purchased from GE-healthcare. Relevant analytical purity grade was purchased from Sinopharm Chemical Reagent Co., Ltd. (Shanghai, China), including NaCI, phenol, sulfuric acid, potassium bromide, hydrochloric acid, anhydrous methanol, anhydrous ethanol, trifluoroacetic acid, PMP reagent, NaOH, acetonitrile, DMSO, iodomethane, formic acid, hydrogen sodium boride, and glacial acetic acid.

### 2.2 Extraction, isolation, and purification of polysaccharides from *S. rugosoannulata*

The plant material was fresh *S. rugosoannulata*, freeze–thaw 30 min (one time) and hot water extracting 30 min (1:100, m/m). The extracts were combined and centrifuged at 3,000 *g* for 10 min, concentrated to 15% of the original volume in a rotary evaporator at 60°C and precipitated overnight in a final concentration of 90% (v/v) ethanol at 4°C. After centrifugation, the separated precipitate was re-dissolved using ultrapure water and deproteinated enzymatically (neutral protease, 37°C, 2 h). Hydrodialysis (MWCO 3000 Da) for 24 h and lyophilization gave the crude polysaccharides (31).

Crude polysaccharides were prepared in distilled water to 0.2 g/ml and fractionated using the DEAE (45 mm × 260 mm) column on AKTA Purea 25 with distilled water and 0–0.5 M NaCl as eluent at a flow rate of 1 ml/min, collecting one tube every 4 min. The total polysaccharides content was determined by phenol-sulfuric acid method, and two fractions (SRF-1 and SRF-2) were extracted and freeze-dried. SRF-1 was identified as the major lipid-lowering fraction by assay and further purified using Sephacryl S-200 HR (26 mm × 1,000 mm) column at a flow rate of 0.4 ml/min with a mobile phase of 0.15 M NaCl solution and a collector for 10 min to collect one tube. The distribution curves of its sugar content were examined and obtain two target polysaccharides, SRF-3 and SRF-4, of which SRF-3 was the major fraction, which were then lyophilized and stored in a dry environment for subsequent studies.

### 2.3 Homogeneity and molecular weight of SRF-3

Distilled water was used as a blank for zeroing and used as a solvent to configure SRF-3 to the 1 mg/ml solution used, then SRF-3 solution was placed in a quartz colorimetric cup and scanned at 190–900 nm at full UV-visible wavelength (UV-2700, Shimadzu, Japan).

High performance gel permeation chromatography (HPGPC, LC-10Avp, Japan) was used to determine the relative molecular mass of SRF-3. The SRF-3 sample solution was prepared at a concentration of 5 mg/ml and dextran was used as a standard, and all samples were filtered using a 0.45 μm filter membrane prior to detection. The chromatographic column was a Shimadzu CLASS-Vp system with a TSK-gel G-3000 PWXL 7.8 × 300 mm, a RID-10A parallax refractive detector, a mobile phase of 0.2 M NaCl aqueous solution, the injection volume of 20 μl with flow rate of 0.6 ml/min and temperature of 40°C.

### 2.4 Monosaccharide composition analysis

The monosaccharide composition of SRF-3 was determined by PMP derivatization combined with ultra-high performance liquid chromatography (UHPLC). The SRF-3 sample was weighed 2 mg, 1 ml of anhydrous methanol solution containing 1 M hydrochloric acid was added, the tube was filled with N_2_ and sealed, hydrolyzed at 80°C for 16 h. After blowing dry with nitrogen, add 1 ml of 2 M trifluoroacetic acid, hydrolyzed at 120°C for 1 h. A small amount of ethanol was added and dried in a water bath at 60°C and repeated 3–5 times to completely evaporate the trifluoroacetic acid. Add 0.5 ml of PMP reagent and 0.3 M NaOH solution to the dried sample obtained after complete acid hydrolysis, and after the sample is fully dissolved, take 0.2 ml of it in a small centrifuge tube, water bath at 70°C for 30 min. After centrifugation at 10,000 *g* for 5 min, add 0.3 M of hydrochloric acid solution 0.1 ml and distilled water 0.1 ml, mix thoroughly. Add 1 ml of dichloromethane, mix well and then extract the remaining PMP reagent, aspirate the dichloromethane layer, retain the aqueous layer and repeat three times (32, 33). The samples were filtered through 0.22 μm membrane and then assayed. The chromatography was performed on a Shimadzu UHPLC system with a Compass C18 (150 × 4.6 mm) column using a mobile phase of PBS (0.1 M, pH 7): acetonitrile 81:19 (v/v) at a flow rate of 1.0 ml/min with a sample volume of 10 μl and a detection wavelength of 245 nm.

### 2.5 Infrared spectroscopy

The dried SRF-3 samples were mixed well with KBr, then pressed into thin slices for FT-IR analysis, then scanned with a PerkinElmer Spectrum Two spectrometer (PerkinElmer, USA) to record FT-IR spectra from 4,000 to 400 cm^–1^. The data were analyzed and processed using OMSNIC spectroscopy software (34).

### 2.6 Structural characterization

#### 2.6.1 Methylation analysis

A total of 10 mg of SRF-3 was dissolved in 1 ml DMSO, 0.5 ml NaOH-DMSO suspension was added and mixed well. Slowly add 1 ml of iodomethane in an ice bath, protected from light, stirring magnetically for 30 min and add 2 ml distilled water to abort the reaction. Dialysis was performed for 24 h each in flowing tap water and distilled water, respectively. After repeating the above steps for the second methylation, the extraction was carried out three times using dichloromethane, followed by reverse extraction using water, blowing the organic phase dry with an air pump, dissolving in distilled water and lyophilizing. The methylated samples were subjected to infrared spectroscopy to examine the methylation. To the above dried sample of methylated sugars, 1 ml of mixed acid (HCOOH:H_2_O:TFA = 3:2:1) was added, sealed with N_2_ and hydrolyzed at 100°C for 6 h. After hydrolysis, anhydrous ethanol was added repeatedly to evaporate the mixed acid to pH = 7 (at temperatures below 40°C). Add 1 ml of 30 mg/ml NaBH_4_ solution at room temperature and stir for 12 h. Neutralize by adding about 100 μl of 50% glacial acetic acid, add an appropriate amount of strong acidic cation exchange resin, stir magnetically for 20 min, filter (remove the resin), add methanol repeatedly to the filtrate and evaporate the boric acid to neutral (temperature below 40°C). Add 0.5 ml each of acetic anhydride and anhydrous pyridine, seal with N_2_ and react at 100°C for 2 h, then the reaction was terminated by adding 1 ml of distilled water to an ice bath, which was sealed for 5 min. A total of 2 ml of dichloromethane and 2 ml of distilled water were added, and the organic phase was reverse extracted three times, the aqueous phase was removed, the organic phase was blown dry, dissolved in 1 ml of chromatographically pure dichloromethane, filtered, and analyzed by Gas Chromatography–Mass Spectrometer (GC-MS). The chromatographic column was an Agilent DB-35 ms with an injection temperature of 300°C and an auxiliary heater temperature of 280°C. The ramp-up procedure: initial temperature 140°C with 2 min retention, 5°C/min to 170°C with 3 min retention, 1°C/min to 180°C with 5 min retention, 3°C/min to 220°C with 1 min retention, 20°C/min to 295°C with 3 min retention.

#### 2.6.2 NMR spectroscopy analysis

A total of 20 mg of dried SRF-3 sample was dissolved in 0.5 ml of D_2_O and left at 20°C for 12 h to dissolve completely. The one-dimensional nuclear magnetic resonance (NMR) spectra (1H NMR, 13C NMR) and two-dimensional NMR spectra (HSQC, HMBC) were measured at 20°C (Bruker Avance 600 MHz, Bruker, Switzerland). 1H NMR was detected at 600 MHz and 13C NMR was detected at 150 MHz.

### 2.7 Antioxidant activity

#### 2.7.1 Hydroxyl radical scavenging capacity

One milliliter sample solution was mixed with an equal volume of ferrous sulfate solution (Fe⋅SO_4_-7H_2_O, 9 mM/L) and hydrogen peroxide solution (10 mM/L). After 10 min of reaction at 37°C, 1 ml of salicylic acid solution (9 mM/L) was added, mixed well and reacted at 37°C for 30 min. Pure water as a blank control, the absorbance of the reaction solution at 510 nm was recorded (35).


$$\begin{array}{l} \text{Hydroxyl radical scavenging capacity (\%)}=\\ \left({\text{A}}_{0}-{\text{A}}_{1}\right)/{\text{A}}_{0}\times 100 \end{array}$$

where A_0_ is the absorbance of control reaction (without addition of the sample), A_1_ was absorbance of sample solution in reaction mixture.

#### 2.7.2 DPPH* radical scavenging capacity

DPPH solution was prepared with anhydrous ethanol at 0.1 mM/L and stored away from light. Two milliliter sample solution was mixed with equal amount of DPPH solution and shaken well and left to stand for 30 min in the dark at 20°C. The DPPH solution was used as a blank control and the absorbance was measured at 520 nm (35).


$$\begin{array}{l} {\text{DPPH}}^{*}\text{ radical scavenging capacity (\%)}=\\ \left({\text{A}}_{0}-{\text{A}}_{1}\right)/{\text{A}}_{0}\times 100 \end{array}$$

where A_0_ is the absorbance of the control reaction (without addition of the sample), A_1_ was the absorbance of sample solution in the reaction mixture.

#### 2.7.3 Lipid peroxidation inhibition capacity

Egg yolk homogenate (10%, v/v) was used as the lipid medium for the reaction. A total of 0.1 ml test solution was mixed with 0.5 ml of egg yolk homogenate, 0.4 ml of pure water and 50 μl of ferrous sulfate solution (Fe⋅SO_4_-7H_2_O, 70 mM/L). After incubation at 37°C for 30 min, 1.5 ml of acetic acid solution (20%, v/v, pH 3.5) and 1.5 ml of thiobarbituric acid solution (0.8% by mass, prepared with 1.1% sodium dodecyl sulfate solution) were added. Five milliliter of n-butanol was added and centrifuged at 5,000 r/min for 15 min. The absorbance of the supernatant was measured at 532 nm (36).


$$\begin{array}{l} \text{Lipid peroxidation inhibition capacity (\%)}=\\ \left({\text{A}}_{0}-{\text{A}}_{1}\right)/{\text{A}}_{0}\times 100 \end{array}$$

where A_0_ is the absorbance of control reaction (without addition of the sample), A_1_ was the absorbance of sample solution in reaction mixture.

### 2.8 Hypolipidemic activities *in vitro*

#### 2.8.1 Cholesterol adsorption capacity

Samples were added to 30 ml of fresh egg yolk solution (10%, v/v), adjusted to pH 7 using NaOH and HCl solution (simulate the environment of intestine), shaken for 2 h at 37°C, centrifuged at 3,000 *g* for 20 min, the supernatant was combined and fixed to 100 ml, then 1 ml was diluted by adding 9 ml of glacial acetic acid, and the cholesterol content in the diluted solution was determined by phthalaldehyde method (2).

#### 2.8.2 Pancreatic lipase inhibitory capacity

The sample was mixed with 3 ml of PBS (0.1 M, pH = 7.4), 0.5 ml of 1.2 mg/ml pancreatic lipase and 0.5 ml of 0.08% p-NPP (p-nitrophenyl phosphate disodium salt), then shake for 30 min at 37°C in a water bath and record the absorbance value at 410 nm, recorded as A_1_. No sample plus lipase solution is denoted as A_0_. Add the sample without lipase solution and write A_2_ (3).


$$\begin{array}{l} \text{Pancreatic lipase inhibitory capacity (\%)}=\\ \left[1-\left({\text{A}}_{1}-{\text{A}}_{2}\right)/{\text{A}}_{0}\right]\times 100\% \end{array}$$

#### 2.8.3 Bile acid salt binding capacity

The sample was taken in a conical flask, 30 mg pepsin and 1 ml of 0.01 M hydrochloric acid solution were added and shaken in a water bath at 37°C for 1 h. The pH of the system was adjusted to 6.3 with NaOH solution, 40 mg trypsin was added and shaken at 37°C for 1 h (simulate the environment of intestinal). Add 4 ml of sodium glycocholate/sodium taurocholate and shake in a water bath at 37°C for 1 h, then centrifuge at 3,000 *g* for 20 min, take 2.5 ml of the supernatant and add 7.5 ml of 60% sulfuric acid and measure the absorbance value at 387 nm (4).

### 2.9 Cellular assays

The toxicity of the polysaccharides to HepG2 cells was determined by measuring cell viability with the CCK-8 kit. HepG2 cells were treated with 1 mM free fatty acid (FFA) mixture (oleic acid/palmitic acid, 2:1) for 24 h (37). A hepatocyte fat accumulation model was established, and three administration concentrations of 25 μg/ml (LD), 100 μg/ml (MD), and 400 μg/ml (HD) were selected to intervene in FFA-induced HepG2 cells based on the results of cytotoxicity assay. Cellular activity, extracellular glutamic-pyruvic transaminase (ALT) content and intracellular triglyceride (TG) levels were measured using CCK-8, ALT and TG kits (Nanjing Jiancheng Institute of Biological Engineering, Nanjing, China), respectively.

### 2.10 Statistical analysis

GraphPad Prism 7.0 was used to perform statistical analysis of the data. The results of the Shapiro–Wilk normality test were used to ensure that the numbers were normally distributed. ANOVA with Tukey’s test was used to assess whether there were any significant differences between the groups. Results were expressed as mean ± SD with at least six biological replicates for each independent experiment. The threshold for statistical significance was set at *p* < 0.05.

## 3. Results

### 3.1 Purification and screening of polysaccharides fractions with hypolipidemic activity

**FIGURE 1:**
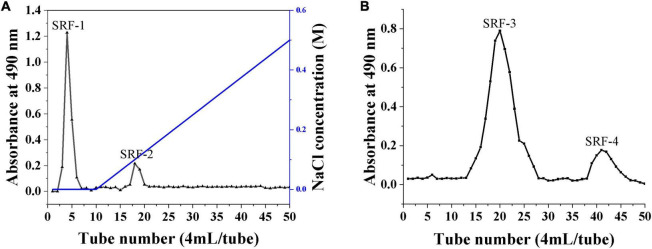
Isolation and purification of polysaccharides from *S. rugosoannulata* by gel column. **(A)** DEAE cellulose column for the separation of crude polysaccharides. **(B)** SRF-1 was further separated by a Sephacryl S-200 high-resolution column.

The crude polysaccharides (SRF) were extracted from *S. rugosoannulata* by freeze–thaw combine with hot water, and purified by ethanol precipitation, protein removal, and freeze-drying steps. Then cellular experiments were conducted to screen the polysaccharides with hypolipidemic efficacy from SRF. HepG2 lipid accumulation model was prepared by FFA-induced, and the lipid-lowering efficacy of SRF-1 and SRF-2 isolated from DEAE-Sepharose Fast Flow columns (Figure 1A) was compared by detecting the cell viability, intracellular TG content and extracellular AST content of FFA-induced HepG2 cells.

**FIGURE 2:**
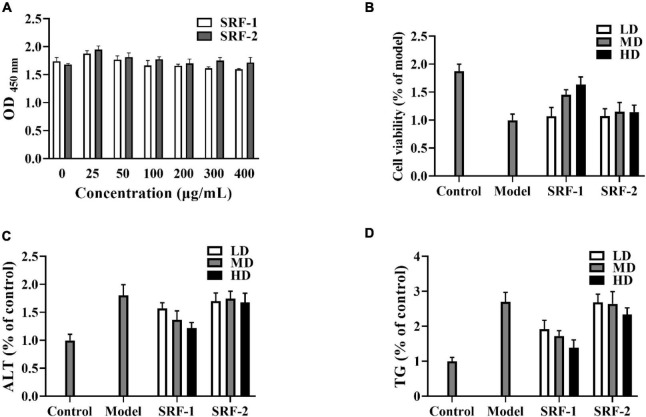
Effects of SRF-1 and SRF-2 on FFA-induced HepG2 cells. **(A)** Cell viability of HepG2 cells. **(B)** Cell viability of FFA-induced HepG2 cells. **(C)** Extracellular ALT levels. **(D)** Intracellular TG levels.

As shown in Figure 2, SRF-1 significantly increased FFA-induced HepG2 cell viability and decreased extracellular ALT content and intracellular TG content, implying that SRF-1 can reduce FFA-induced hepatocyte injury and lipid accumulation. Therefore, we chose Sephacryl S-200 HR ion exchange column for further purification and study of SRF-1. As shown in Figures 1B, a 0.15 M NaCl solution eluted SRF-1, and two fractions, named SRF-3 and SRF-4, were obtained, with SRF-3 as the main fraction. Therefore, the present study focused on the structure and activity of SRF-3.

**FIGURE 3:**
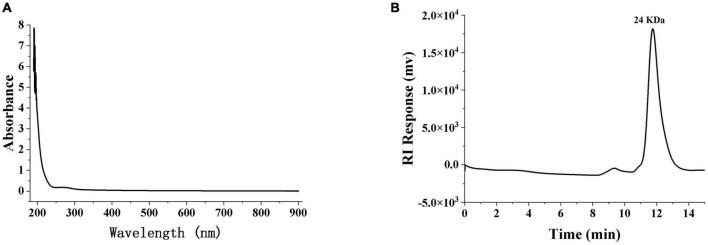
The ultraviolet spectroscopy **(A)** and HPGPC **(B)** of SRF-3. SPF-3 was configured as a 1 mg/ml solution, samples were scanned in a quartz colorimetric cup at 190–900 nm for UV-vis full wavelength, and zeroed with distilled water as a blank.

The purity and average relative molecular mass of SRF-3 were further determined by HPGPC. The HPGPC spectrum of SRF-3 was a sharp and symmetric single elution peak, indicating that SRF-3 was a relatively homogeneous fraction (Figure 3B). The average relative molecular mass of SRF-3 was 24 kDa.

### 3.2 Monosaccharide composition analysis of SRF-3

The phenol-sulfate method was used to determine the total sugar content, while the BCA method was used to determine the protein content. The results showed that SRF-3 contained 95.43% total sugars and 0.2% protein (Table 1). Scanning of the SRF-3 using full wavelength UV spectroscopy showed that SRF-3 had no absorption peaks at 260–280 nm, indicating that SRF-3 was almost free of protein and nucleic acids (Figure 3A).

**TABLE 1:** Component characterization of SRF-3.

Index	SRF-3
Total sugar content (%)	95.43 ± 2.84
Protein content (%)	0.2 ± 0.04
Monosaccharide composition (molar ratio)	
Mannose	0.16
Glucose	0.12
Galactose	0.58
Methyl galactose	0.12
Fucose	0.02

**FIGURE 4:**
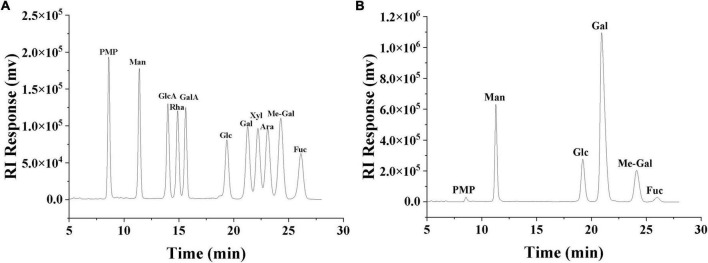
HPLC chromatogram of monosaccharide standards **(A)**. HPLC chromatogram of the monosaccharide composition of SRF-3 **(B)**.

The primary structure of polysaccharides determines the secondary, tertiary and even quaternary structure of polysaccharides, which in turn influences the physicochemical properties and biological activity of polysaccharides (38). The monosaccharide composition of SRF-3 was determined by PMP derivatization combined with UHPLC, by comparing the retention time and peak area with each monosaccharide standard (Figure 4 and Table 1), it was determined that SRF-3 consisted of mannose, glucose, galactose, and methyl galactose in a molar ratio of 8:12:58:12.

Currently, a wide variety of polysaccharides are extracted from edible mushrooms. Liang et al. reported that the PGP-1c extracted from the *Pleurotus geesteranus* was composed of galactose (36.4%), 3-O-methylgalactose (20.8%), mannose (20.7%), glucose (19.9%), and fucose (2.2%) (39). Chen et al. reported that the polysaccharides EP-1 extracted from *Pleurotus eryngii* consisted of D-Glc, D-Gal, and D-Man in the molar ratio: 96.39:2.26:1.35 (11). Manna et al. reported that the polysaccharides extracted from the *L. edodes* consisted of a (1→6)-linked galactose group, a (1→6)-linked (1→3,6)-linked glucose residue, and a terminal pyranose groups in a molar ratio close to 3:1:1:1, respectively (40). The high content of galactose, mannose and glucose in edible mushroom polysaccharides and the agreement of SRF-3 to contain these three monosaccharides, but with a higher content of galactose, indicating that SRF-3 is very different from other typical edible mushroom polysaccharides, which may be related to the properties of the *S. rugosoannulata* itself or the method of polysaccharides extraction.

### 3.3 Structure of SRF-3

#### 3.3.1 FT-IR spectroscopy

**FIGURE 5:**
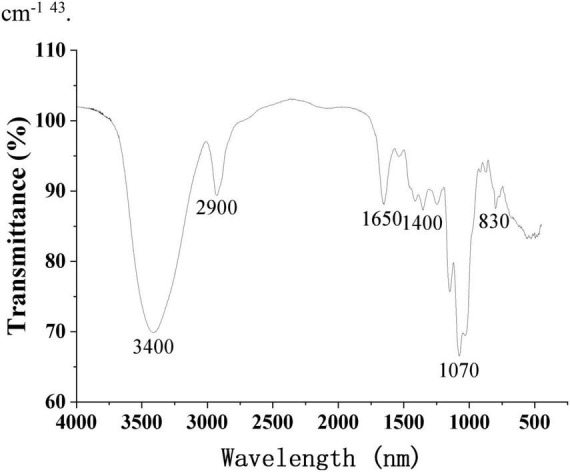
FT-IR spectrum of SRF-3. OMNIC spectral analysis software was used to perform analysis and processing of FT-IR spectral data.

Figure 5 shows the FT-IR spectra of purified SRF-3 from 1,650 to 4,000 cm^–1^. The sample exhibits a distinct polysaccharides characteristic absorption peak with a broad and strong absorption peak of -OH stretching vibration detected near 3,400 cm^–1^. A narrow and weak C-H stretching vibration absorption peak was detected near 2,900 cm^–1^, and a bending vibration absorption peak of OH was shown near 1,650 cm^–1^. In addition, a weak C-H deformation vibration peak was detected around 1,400 cm^–1^, as well as a clear pyran ring absorption peak near 1,070 cm^–1^, and C-H variable angle vibration of the differential isomerization of the α-terminus of pyranose at 855–810 cm^–1^ (40).

#### 3.3.2 Methylation analysis

**FIGURE 6:**
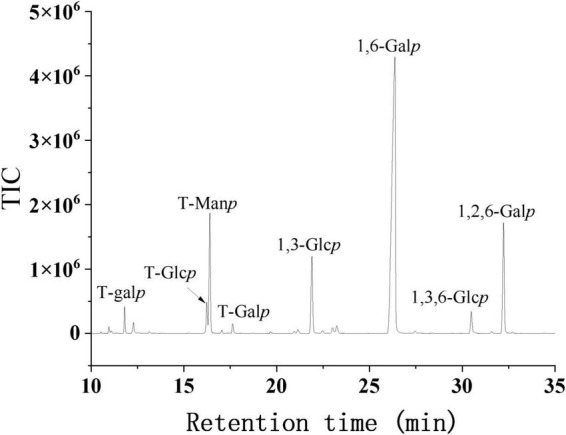
The TIC profiles of the methylated alditol acetylates of SRF-3.

Methylation analysis is the complete methylation of free hydroxyl groups in polysaccharides and is often used to determine the glycosyl bonds and thus resolve the structure of polysaccharides. The methylation, hydrolysis, reduction and acetylation of SRF-3 were carried out and the type of glycosidic bond was determined by GC-MS analysis. The methylation results of SRF-3 are shown in Figure 6 and Table 2. SRF-3 contains mainly 1,6-Gal (60.3%) and 1,2,6-Gal (11.0%), t-Man (10.4%) and t-Fuc (1.6%) were detected indicating that they were mainly present in the non-reducing end form. It is assumed that this homogeneous component is mainly 1,6-Gal as the main chain with substitution at the O-2 position of Gal with a branching degree of 15.4%, and the side chain structure is dominated by t-Man with a small amount of t-Fuc present, indicating that this homogeneous component is a rockweed mannogalactan. In addition, a small amount of 1,3-Glc, 1,6-Glc and 1,3,6-Glc indicated that a small amount of glucan structure was also present in this sample (39).

**TABLE 2:** Results of SRF-3 methylation analysis.

Retention time (min)	PMAAs	Linkage	Molar ratios
11.801	1,5-Di-O-acetyl-1-deuterio-6-deoxy-2,3,4-tri-O-methyl-L-galactitol	L-Fuc*p*-(→1	1.6
16.232	1,5-Di-O-acetyl-1-deuterio-2,3,4,6-tetra-O-methyl-D-glucitol	Glc*p*-(→1	2.5
16.398	1,5-Di-O-acetyl-1-deuterio-2,3,4,6-tetra-O-methyl-D-mannitol	Man*p*-(→1	10.4
17.633	1,5-Di-O-acetyl-1-deuterio-2,3,4,6-tetra-O-methyl-D-galactitol	Gal*p*-(→1	0.9
21.904	1,3,5-Tri-O-acetyl-1-deuterio-2,4,6-tri-O-methyl-D-glucitol	→3)-Glc*p*-(→1	8.3
26.371	1,5,6-Tri-O-acetyl-1-deuterio-2,3,4-tri-O-methyl-D-galactitol	→6)-Gal*p*-(→1	60.3
30.491	1,3,5,6-Tetra-O-acetyl-1-deuterio-2,4-di-O-methyl-D-glucitol	→3,6)-Glc*p*-(→1	2.4
32.229	1,2,5,6-Tetra-O-acetyl-1-deuterio-3,4-di-O-methyl-D-galactitol	→2,6)-Gal*p*-(→1	11.0

#### 3.3.3 NMR analysis

**FIGURE 7:**
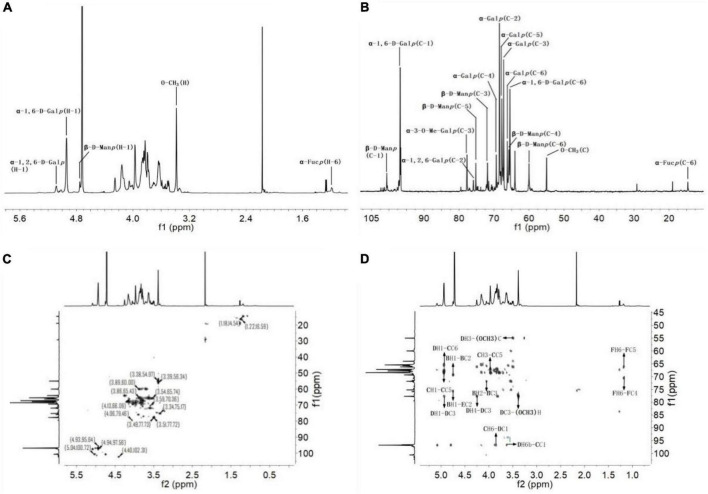
Nuclear magnetic resonance analysis of SRF-3. **(A)** 1H NMR spectrum. **(B)** 13C NMR spectrum. **(C)** HSQC spectrum. **(D)** HMBC spectrum.

Nuclear magnetic resonance is one of the commonly used methods to analyze the structure of polysaccharides and can provide accurate structural information of polysaccharides (41). In the present study, the fine structure of SRF-3 was analyzed using one-dimensional NMR (1H and 13C) and two-dimensional NMR (HSQC and HMBC), and the results are shown in Figure 7 and Tables 3, 4. In the 1H-NMR spectrum, 4.93 ppm is attributed to the heterohead proton signal peaks of α-D-1,6-Gal*p* and α-D-1,6-Me-Gal*p* sugar residues, the signal peak located at 3.38 ppm is the signal peak of H in O-CH_3_, the heterohead proton signal peak of β-D-Man*p* appears at 4.75 ppm. In the 13C-NMR spectrum, the heterohead carbon of α-D-1,6-Gal*p* or α-D-1,6-Me-Gal*p* and α-D-1,2,6-Gal*p* are located at 96.79 and 97.35 ppm, respectively. The signal peak at 75.87 ppm located in the low field is attributed to the C-2 position of α-D-1,2,6-Gal*p*, the signal peak of C in O-CH_3_ peak is located at 54.98 ppm, the hetero-headed carbon signal peak of β-D-Man*p* appears at 100.65 ppm, and the hetero-headed carbon signal peak of β-D-Glc*p* appears at 102.24 ppm (34). Other chemical shifts and connections were attributed by HSQC and HMBC.

**TABLE 3:** 1H and 13C NMR spectra assignments for SRF-3 (ppm).

Sugar residues		1	2	3	4	5	6	O-CH_3_
(A)β-1,6-D-Glc*p*	H	4.41	3.22	3.42	3.34	3.42	3.97, 3.83	–
	C	102.24	73.79	74.61	68.56	75.42	69.12	–
(B)t-β-D-Man*p*	H	4.75	4.05	3.61	3.54	3.34	3.86, 3.69	–
	C	100.65	70.22	71.83	65.73	75.17	59.99	–
(C)α-1,6-D-Gal*p*	H	4.93	3.84	3.97	4.03	4.15	3.85, 3.63	–
	C	96.79	68.45	67.22	69.33	67.76	66.21	–
(D)α-1,6-D-3-Me-Gal*p*	H	4.93	3.84	3.51	4.25	4.15	3.85, 3.63	3.38
	C	96.79	68.45	77.72	65.44	67.76	66.21	54.98
(E)α-1,2,6-D-Gal*p*	H	5.08	3.91	3.97	4.05	4.15	3.85, 3.51	–
	C	97.35	75.87	67.22	69.33	67.76	64.01	–
(F)t-α-L-Fuc*p*	H	5.03	3.56	4.02	3.78	4.12	1.18	–
	C	101.6	71.54	67.48	70.64	66.23	14.63	–

**TABLE 4:** HMBC spectrum chemical shifts of SRF-3.

Sugar residues	H-6/C-6	H-3/C-3	H-1/C-1	Coupling relationship		
				δ H/δ C	Residue	Atom
(B)t-β-D-Man*p*			4.75	69.33	B	C2
				75.87	E	C2
		71.83		4.05	B	H2
(C)α-1,6-D-Gal*p*			4.93	66.21	D	C6
				67.76	C	C5
		4.97		67.76	C	C5
			96.79	3.85	D	H6a
				3.63	D	H6b
(D)α-1,6-D-3-Me-Gal*p*			4.93	66.21	C	C6
				67.76	D	C5
				77.72	D	C3
		3.51		54.98	O-CH_3_	C
		77.72		3.38	O-CH_3_	H
				4.25	D	H4
			97.56	3.85	C	H6a
				3.63	C	H6b
(F)t-α-L-Fuc*p*	1.18			70.64	F	C4
				66.23	F	C5

In HSQC, the H-1/C-1 to H-6/C-6 of Gal, Glc and Man were attributed sequentially, which revealed the presence of α-D-1,6-Gal*p*, α-D-1,6-Me-Gal*p* and α-D-1,2,6-Gal*p* in the sample, as well as a small amount of β-1,6-D-Glc*p* composed of Glc and Man consisting of t-β-D-Man*p*, named below with the letters A, B, C, D, and E, respectively. The linkage order between the individual sugar residues was analyzed by the long-range coupling correlation spectrum HMBC: BH-1/EC-2, CH-1/DC-6, DC-1/CH-6a/6b, CC-1/DH-6a/6b, DH-1/CC-6, DC3-O-CH_3_H, DH3-O-CH_3_C, FH-6/FC-4, FH-6/FC-5. In summary, we can obtain the structural characteristics of this sample as follows: α-D-1,6-Gal*p* and α-D-1,6-Me-Gal*p* are linked to form the main chain structure, with a branch at the O-2 position of Gal, and a small amount of t-β-D-Man*p* and a trace amount of t-α-L-Fuc*p* structure in the form of side chains, which is the rockweed mannogalactan (39).

### 3.4 *In vitro* antioxidant activity of SRF-3

**FIGURE 8:**
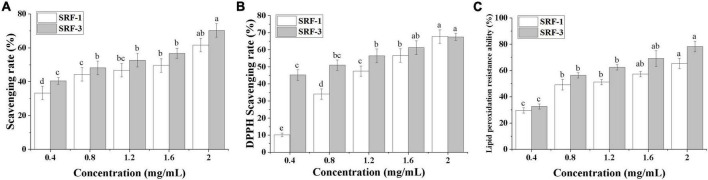
*In vitro* determination of the antioxidant capacity. **(A)** Hydroxyl radical scavenging capacity. **(B)** DPPH scavenging capacity. **(C)** Lipid peroxidation resistance capacity. Values with different letters (a–d) were significantly different (*p* < 0.05).

The antioxidant capacity of SRF-3 was evaluated by three complementary tests on hydroxyl and DPPH radical scavenging capacity and lipid peroxidation inhibition. Scavenging of free radicals is considered to be one of the main mechanisms by which antioxidants slow down the oxidative process, while inhibition of lipid peroxidation can directly reduce the oxidative damage caused by fat accumulation. As shown in Figure 8, the antioxidant capacity of SRF-1 and SRF-3 showed a significant positive correlation with their concentrations.

The scavenging rates of hydroxyl radicals by SRF-1 and SRF-3 were 60.84 and 70.35% at 2 mg/ml, respectively. The IC_50_ values of SRF-1 and SRF-3 were 1.62 ± 0.02 and 0.96 ± 0.04 mg/ml, respectively (Figure 8A). The DPPH scavenging activities of SRF-1 and SRF-3 were significantly different, with 10.22 and 45.22% scavenging of DPPH radicals at low concentrations (0.4 mg/ml) for SRF-1 and SRF-3, respectively. The IC_50_ was 1.32 ± 0.05 and 0.80 ± 0.03 mg/ml, respectively. But at a concentration of 5 mg/ml, both DPPH radical scavenging rates were similar (Figure 8B). At low concentrations, there was no significant difference in the lipid peroxidation inhibitory capacity of SRF-1 and SRF-3, but as the concentration increased, the lipid peroxidation inhibitory capacity of SRF-3 was significantly higher than that of SRF-1, and at 2 mg/ml, the inhibitory capacity of SRF-1 and SRF-3 lipid peroxidation was 65.34 and 78.25%, respectively. The IC_50_ values of SRF-1 and SRF-3 were 0.81 ± 0.01 and 0.69 ± 0.02, respectively (Figure 8C). Overall, the antioxidant activity of SRF-3 was significantly higher than that of SRF-1, predicting that SRF-3 may be the main antioxidant component of SRF.

### 3.5 *In vitro* hypolipidemic effects of SRF-3

**FIGURE 9:**
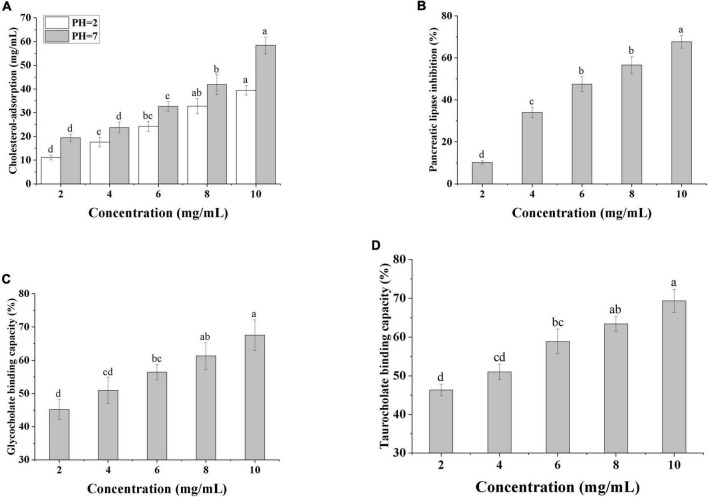
*In vitro* determination of the hypolipidemic capacity. **(A)** Cholesterol-adsorption capacities of SRF-3. **(B)** Pancreatic Lipase inhibition capacity of SRF-3. **(C)** Glycocholate binding capacity of SRF-3. **(D)** Taurocholate binding capacity of SRF-3. Values with different letters (a–d) were significantly different (*p* < 0.05).

Numerous studies have shown that polysaccharides can modulate blood lipids in a variety of ways, including lowering serum total cholesterol (TC), low-density lipoprotein cholesterol (LDL-C), and increasing bile acid efflux (42). The *in vitro* lipid-lowering ability of SRF-3 was verified by testing the cholesterol adsorption ability, pancreatic lipase inhibition ability, and bile acid salt binding ability of SRF-3. As seen in Figure 9A, the adsorption capacity of SRF-3 for cholesterol adsorption showed a positive correlation with its concentration, and the cholesterol adsorption capacity was higher at pH 7 than pH 2 at the same concentration, and the cholesterol binding capacity of SRF-3 reached 60% at 10 mg/ml in the pH 7 environment, indicating that the cholesterol adsorption capacity of SRF-3 was higher in the intestinal environment than in the gastric environment. The small intestine is an important pathway for the biological organism to obtain exogenous cholesterol from food, it can be judged that SRF-3 can reduce blood lipids by affecting the absorption of exogenous cholesterol by the biological organism. Figure 9B shows that as the concentration of SRF-3 increases, its inhibition of pancreatic lipase activity is enhanced, implying that SRF-3 has some potential in reducing exogenous fat digestion. Figures 9C, D show the binding ability of SRF-3 for glycocholate and taurocholate, respectively, and the results indicate that the bile acid salt binding ability also showed a significant positive correlation with SRF-3 concentration, implying that SRF-3 can reduce the circulation of bile acids in the body by binding bile acid salt in bile acids, thus promoting the excretion of lipids and lowering blood lipids (6).

## 4 Discussion and conclusion

In this article, two crude polysaccharides fractions, SRF-1 and SRF-2, were isolated from *S. rugosoannulata* by DEAE Sepharose Fast Flow chromatography, and SRF-1 was identified as an effective lipid-lowering fraction by cellular assay, and then Sephacryl S-200 high resolution column Chromatography was used to separate and purify to obtain the fractions SRF-3 and SRF-4, with SRF-3 being the major fraction. SRF-3 was characterized by UHPLC, acid hydrolysis, methylation and one/two-dimensional NMR, and its hypolipidemic potential was evaluated by *in vitro* experiments. The results showed that SRF-3 consists of five monosaccharides (Rha, Ara, Man, Glc, and Gal) with an average weight of 24 kDa. Structural analysis showed that SRF-3 consists of α-D-1,6-Gal*p* and α-D-1,6-Me-Gal*p* linked to form the main chain structure, with a branch at the O-2 position of Gal with 15.4% branching, and a small amount of t-β-D-Man*p* and a trace amount of t-α-L-Fuc*p* are present as side chains. The production and removal of reactive oxygen species in living organisms is in a dynamic equilibrium, and lipid peroxidation resulting from fat accumulation can upset this equilibrium, thus allowing the concentration of reactive oxygen species to exceed physiological limits and causing damage to the body’s cells. Therefore, lipid-lowering ingredients must not only have lipid-lowering effects, but also have the ability to reduce the damage caused by lipid accumulation in the organism. SRF-3 exhibited free radical scavenging ability in antioxidant assays *in vitro*. This phenomenon also occurs in other edible fungal polysaccharides. Zhang et al. (35) isolated three crude polysaccharides from *Agaricus blazei* that also showed good antioxidant activity and concentration-dependent properties. Liu et al. (43) isolated water-soluble polysaccharides and alkali-soluble polysaccharides from the *Oudemansiella radiata*, whose main components were mannose, glucose, and galactose, and showed good antioxidant activity and antioxidant activity with concentration dependence. Through *in vitro* lipid-lowering experiments, we speculate that SRF-3 can reduce the intake and accumulation of lipids *in vivo* by binding exogenous cholesterol, reducing the digestion and decomposition of exogenous lipids by lipase and binding bile acid salts in bile acids (4). Our results suggest that SRF-3 has potential as a raw material for a lipid-lowering drug.

The structure and biological activity of polysaccharides in mushrooms are diverse and the biological activity of mushroom polysaccharides depends on the molecular weight of the polysaccharide, the monosaccharide composition, the degree of branching and the type of glycosidic bond. Previous investigations have revealed that mushroom polysaccharides such as *Auricularia auricula*, *A. blazei*, *Agaricus bisporus*, *Cordyceps sinensis*, *Grifola frondosa*, *Ganoderma lucidum*, *Morchella esculenta*, *Pleurotus eryngii*, *Pleurotus ostreatus*, *Pholiota nameko*, and *Collybia albuminosa* both have a substantial influence on antioxidant, anti-inflammatory, immunomodulatory, hypoglycemic, and hypolipidaemic effects (44–51). Liu et al. (30) used macroporous adsorption resin and ion exchange chromatography to isolate two structurally different glucose-based polysaccharides, SRP-1 and SRP-2, from *S. rugosoannulata*. Both polysaccharides contained a (1→, 6)-α-D-glucan backbone, but the monosaccharides of SRP-1 and SRP-2 molar ratios and glycosidic bond types were different, and both polysaccharides exhibited antioxidant activity. Their extraction and elution methods were similar to our study, but the extracted polysaccharides differed significantly in structure from those obtained by our isolation. Liang et al. (39) isolated a glucomannan galactan with a glycan chain structure similar to SRP-3 from *P. ostreatus*, with α-(1→6)Gal as the main chain and β-D-Man*p*-(1→, →3)-α-D-Glc*p*-(1→ and a small amount of Fuc*p* as the side chain, and *in vitro* experiments showed that the polysaccharides has immunomodulatory activity. Ge et al. (52) isolated a mannogalactan composed of glucose, galactose, and mannose from *Helvella leucopus*, which showed significant lipid-lowering activity in high-fat fed mice. We suggest that the monosaccharide composition of glucose, galactose, and mannose and the main chain of (1→, 6)-α-D-glucan may be responsible for the hypolipidemic and antioxidant effects of SRF-3, but further experiments are needed to verify this.

Different extraction and isolation methods may result in differences in the structure and activity of polysaccharides. In this study, SRF-3 was isolated and purified by DEAE Sepharose Fast Flow and Sephacryl S-200 high-resolution column chromatography, and its chemical structure and lipid-lowering activity were investigated, which could be useful for its future application development in lipid-lowering. However, the biological activity of polysaccharides depends not only on their structure and molecular size, but also on their conformation. Therefore, additional conformational analyses of the polysaccharides of *S. rugosoannulata* are needed in the future.

## Data availability statement

The original contributions presented in this study are included in the article/supplementary material, further inquiries can be directed to the corresponding authors.

## Author contributions

DW and YZ established the research direction. TL, HF, and HL guided the thesis writing. YG carried out the experiments and wrote the manuscript. SZ helped to consult the literature. GA, CY, XT, and CB assisted in experimental operation and data recording. All authors contributed to the article and approved the submitted version.
